# Multivariate analysis of the relationship between gluteal muscle contracture and coxa valga

**DOI:** 10.1186/s12891-021-04447-7

**Published:** 2021-06-19

**Authors:** Yi Zhang, Qihang Su, Yuanzhen Zhang, Heng’an Ge, Wang Wei, Biao Cheng

**Affiliations:** 1 Department of Orthopaedics, Shanghai Tenth People’s Hospital, School of Medicine, Tongji University, Shanghai, 200092 China; 2 First Department of Orthopaedics, The Second Affiliated Hospital of Xi’an Jiaotong University, No. 157 Xiwu Road, Xi’an, 710004 Shaanxi China

**Keywords:** Gluteal muscle contracture, Gluteal fibrosis, Coxa valga, Neck-shaft angle, Sacro-femoral-pubic angle, Pelvic tilt

## Abstract

**Background:**

Gluteal muscle contracture (GMC) is a disease characterized by the limited function of the hip joint, knee pain, and abnormal gait. There is a lack of research on the effect of GMC on the hip joint structure to date. This study aims to analyze the association between GMC and the deformity of the hip and pelvis.

**Methods:**

Standing anteroposterior pelvic radiographs of 214 patients (152 with gluteal muscle contracture and 62 without gluteal muscle contracture) were retrospectively collected. Neck–shaft angle, lateral center edge angle, Tönnis angle, femoral head coverage index, acetabular depth, Sacro-femoral-pubic angle, and obturator foramen ratio were respectively measured and included in the following statistical analysis. The collected data were analyzed using logistical regression and multiple linear regression to explore the factors influencing coxa valga and SFP angle.

**Results:**

GMC was identified as a common factor significantly associated with coxa valga and increased SFP angle. There is a difference of risk factors in logistic regression for coxa valga between the left and right sides.

**Conclusion:**

GMC is a significant risk factor for coxa valga and increased SFP angle. Given that GMC can cause coxa valga and likely alter the pelvis’s position, GMC should be paid attention to and treated early.

## Introduction

Gluteal muscle contracture (GMC), also known as gluteal fibrosis, is a common condition characterized by the limited function of the hip joint, knee pain, and abnormal gait [1–4]. GMC mainly caused by contracture of tensor fascia lata, iliotibial band, gluteal muscles, and the relevant fascia tissue, leading to abnormal clinical manifestation including snapping hip, frog-leg posture (unable to adduct hip in squatting position), inability to sit with crossed legs, skin dimple of buttock, and contracture stripe [3, 5]. Although many researchers initially considered GMC as sporadic, it was found to be relatively common in lots of countries, especially in Chinese [6]. In the last several decades, due to the wide application of penicillin gluteal intramuscular injection with benzyl alcohol in economically underdeveloped areas, many Chinese people get GMC in childhood and suffer from following squatting inconvenience and knee pain caused by GMC [3, 7].

In the existing literature on GMC, most studies have only focused on the treatment method rather than the influence of GMC on hip joint development [4, 5, 8–13]. Several researchers had briefly mentioned that GMC might result in secondary coxa valga and pelvic tilt [4, 5, 13]. However, to date, no study has been performed to investigate the exact effect of GMC on the secondary deformity of the pelvis and hip joint. Only a few previous studies have investigated radiographic manifestations of GMC, including an “iliac hyperdense line” on the ilium [13, 14]. However, they failed to effectively evaluate the influence of GMC on the hip structure that could lead to pathological biomechanical changes [13, 14]. Much uncertainty still exists about the association between GMC and the hip structure.

This paper aims to analyze the association between GMC and the deformity of the hip and pelvis. This study will improve our understanding of changes secondary to GMC and provide evidence for treatment intervention timing.

## Methods

### Patients selection

This study was approved by the Ethics Committee of Shanghai Tenth People’s Hospital affiliated to Tongji University. The procedures used in this study adhere to the tenets of the Declaration of Helsinki. The study retrospectively collected patients, with or without GMC, who received standing anteroposterior (AP) pelvic X-rays for clinical or research purposes at the same institution between October 2019 and February 2021. Informed consent was obtained from all individual participants (or their legal guardians for children under 18) in the study.

Inclusion criteria for GMC patients were: (1) patients diagnosed as GMC by clinical history, physical examination, and intraoperative arthroscopic observation; (2) age between 15 years and 50 years; (3) no history of trauma or surgery (e.g., hip, lower extremities, spine) that affect the structure of the hip; (4) no history of cerebral palsy, scoliosis, congenital deformities, and other diseases affect the hip structure.

Inclusion criteria for patients without GMC were: (1) age between 15 years and 50 years; (2) no history of trauma or surgery (e.g., hip, lower extremities, spine) that affect the structure of the hip; (3) no history of cerebral palsy, scoliosis, congenital deformities and other diseases that affect hip structure; (4) no history of repeated gluteal intramuscular injection. (5) no symptoms or signs similar to GMC.

### Radiographic parameter

Preoperative standard anteroposterior pelvic radiographs were obtained. Neck–shaft angle (NSA) [15], lateral center edge angle (LCEA) [16], Tönnis angle [17], femoral head coverage index (FHCI) [18], acetabular depth [19, 20], Sacro-femoral-pubic(SFP) angle [21] and obturator foramen ratio (OF ratio) [22] were respectively measured by an experienced surgeon and a radiologist using previous research methods. All measurements were performed on both left and right sides of the pelvis, and the data were collected.

**Fig. 1:**
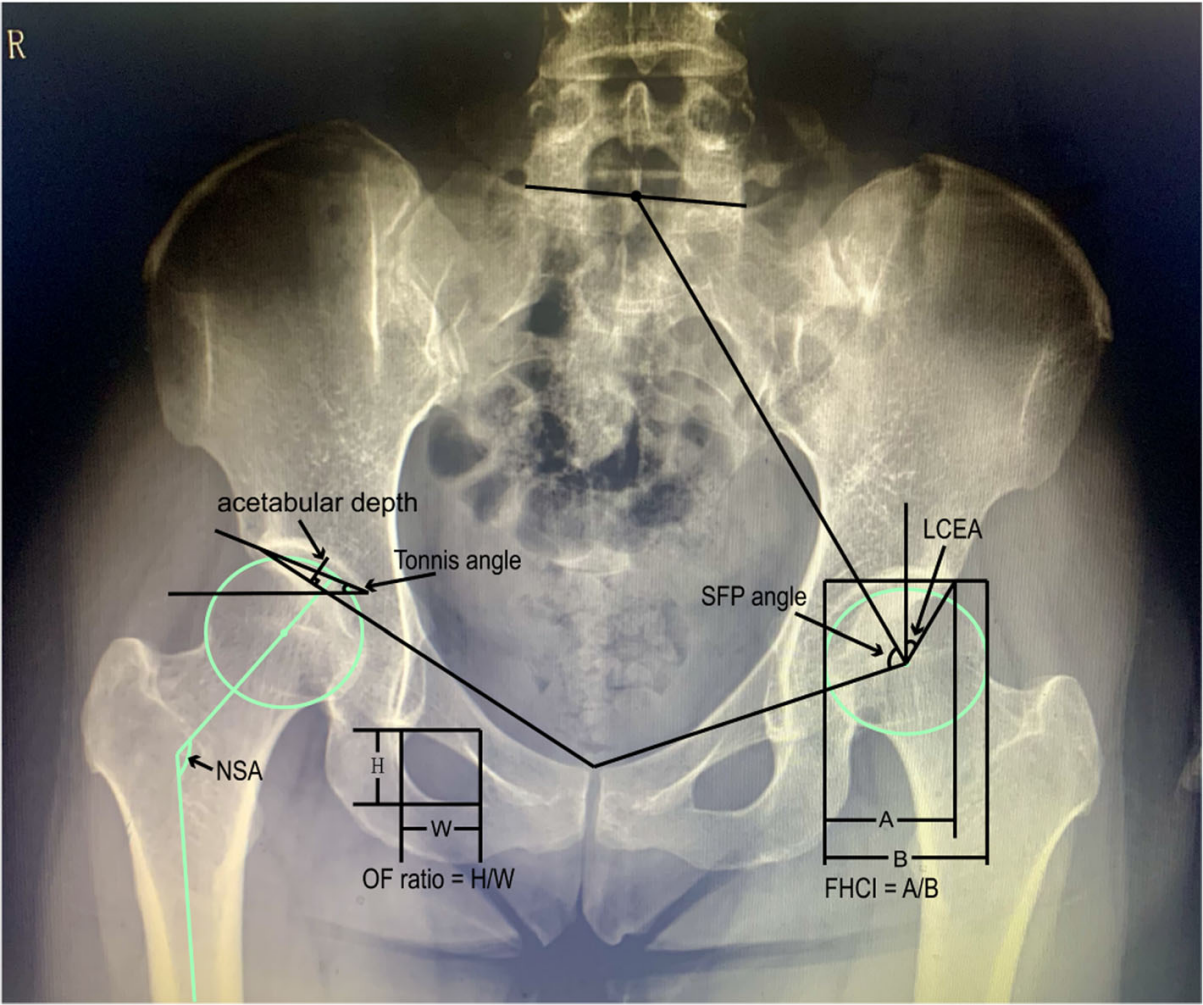
Parameter measurement in anteroposterior pelvic radiographs. NSA, neck-shaft angle; LCEA, lateral center edge angle; FHCI, femoral head coverage index; SFP angle, Sacro-femoral-pubic angle; OF ratio, obturator foramen ratio

The hip and pelvis measurements were performed on the AP pelvic radiographs, as shown below (Fig. 1).

1. NSA: the angle between the long axes of the femoral neck and the femoral shaft.

2. LCEA: the angle between the line connecting the center of the femoral head to the lateral edge of the acetabular roof and the line perpendicular to the pelvic horizontal.

3. Tönnis angle: the angle between the line connecting the medial sourcil margin and lateral sourcil margin and the pelvis’s horizontal axis.

#### 4. FHCI: the quotient of the horizontal distance from the medial femoral cortex to the acetabulum’s lateral edge divided by the femoral head’s total horizontal width

5. acetabular depth: The maximum vertical distance from the acetabular roof to the line connecting the ipsilateral lateral edge of the acetabular roof and the symphysis pubis’s superior margin.

6. SFP angle: the angle between the midpoint of the upper sacral endplate, the center of femoral head and the upper midpoint of the symphysis pubis.

#### 7. OF ratio: the obturator foramen’s maximum vertical height divided by the maximum horizontal width

### Statistical analysis

Univariate logistic regression analysis was performed to screen risk factors for coxa valga. All *P* values were two-sided, and risk factors with *P* < 0.20 in univariate analysis were included in a multivariate analysis. Multivariate logistic regression was performed to identify independent risk factors, and a stepwise method was used to identify the valuable combination of factors that could most precisely predict coxa valga. Multiple linear regression analysis was used to explore the factors influencing SFP angle with a stepwise method. Chi square test and t-test were used to verify the characteristics differences between the GMC patients with or without coxa valga deformity. All data were analyzed using the SPSS software (version 12.0, IBM, Chicago, USA). *P* < 0.05 were considered to be statistically significant.

## Results

### Patient demographics

A total of 214 patients (428 hips) were included in the analysis. Among them, 152 (83 women and 64 men) patients were diagnosed as GMC and underwent arthroscopic tight fibrous band release in our hospital by the senior author. Characteristics and classification of the patients are shown in Tables 1 and 2. According to the imaging diagnostic criteria proposed in the previous research [15], 115 hips (58 left, 57 right) were diagnosed as coxa valgus, and no hip was diagnosed as coxa varus.

**Table 1:** Patients basic characteristics

	Mean [SD]	n (percent)
Age (years)	31.39 [6.58]	
< 30	24.72 [3.67]	77 (36.0%)
30–39	33.83 [2.86]	120 (56.1%)
≥ 40	44.29 [4.22]	17 (7.9%)
Gender		
male		101 (47.2%)
female		113 (52.8%)
BMI (kg/m^2^)	22.37 [3.46]	
< 18.5	17.40 [0.90]	30 (14.0%)
18.5–25	21.88 [1.85]	140 (65.4%)
> 25	27.33 [2.12]	44 (20.6%)
GMC		
-		62 (29%)
+		152 (71%)

*SD* standard deviation, *BMI* body mass index, *GMC* gluteal muscle contracture, − patients without GMC, + patients with GMC

**Table 2:** Imaging characteristics of the study patients

	Left side		Right side	
	Mean [SD]	n (percent)	Mean [SD]	n (percent)
NSA	135.66 [9.40]		135.33 [8.54]	
114°-140°	131.30 [5.51]	156 (72.9%)	131.44 [5.63]	157 (73.3%)
> 140°	147.37 [7.49]	58 (27.1%)	146.06 [5.46]	57 (26.7%)
LCEA	36.99 [6.65]		35.22 [7.05]	
< 25°	22.72 [1.62]	5 (2.3%)	21.62 [3.29]	6 (2.8%)
25°-40°	34.01 [3.57]	145 (67.8%)	32.72 [4.08]	158 (73.8%)
> 40°	44.86 [4.58]	64 (29.9%)	44.74 [5.15]	50 (23.4%)
Tönnis angle	6.11 [5.11]		7.44 [5.49]	
< 0°	−3.70 [2.46]	26 (12.1%)	−4.11 [3.25]	16 (7.5%)
0°-10°	5.80 [2.43]	140 (65.4%)	5.71 [2.59]	126 (58.9%)
> 10°	12.33 [2.23]	48 (22.4%)	13.05 [2.99]	72 (33.6%)
FHCI	0.86 [0.05]		0.86 [0.06]	
< 0.75	0.74 [0.01]	3 (1.4%)	0.72 [0.03]	11 (5.1%)
≥ 0.75	0.86 [0.05]	211 (98.6%)	0.87 [0.05]	203 (94.9%)
acetabular depth	1.41 [0.33]		1.33 [0.31]	
< 0.9	0.81 [0.09]	13 (6.1%)	0.80 [0.08]	16 (7.5%)
≥ 0.9	1.46 [0.30]	201 (93.9%)	1.37 [0.28]	198 (92.5%)
SFP angle	70.07 [7.25]		70.34 [7.02]	
< 60°	56.64 [3.36]	19 (9.9%)	56.04 [3.66]	16 (7.5%)
60°-80°	70.01 [5.03]	175 (81.8%)	70.26 [4.68]	179 (83.6%)
> 80°	82.57 [1.69]	20 (9.3%)	83.09 [1.35]	19 (8.9%)
OF ratio	1.01 [0.25]		1.00 [0.26]	
< 0.7	0.63 [0.07]	17 (7.9%)	0.59 [0.14]	18 (8.4%)
0.7–1.4	0.99 [0.17]	179 (83.6%)	0.99 [0.17]	178 (83.2%)
> 1.4	1.55 [0.15]	18 (8.4%)	1.57 [0.15]	18 (8.4%)

*SD* standard deviation, *NSA* Neck–shaft angle, *LCEA* lateral center edge angle, *FHCI* femoral head coverage index, *SFP angle* Sacro-femoral-pubic angle, *OF ratio* obturator foramen ratio

### Logistic regression for coxa Valga

In univariate regression analysis, the association between coxa valga and all factors was respectively investigated according to the left and right sides. On the left side, the *P* values of GMC, Tönnis angle, and OF ratio were less than 0.2, and these factors were included in the subsequent step analysis. (Table 3) In the same way, age, weight, height, GMC, femoral head coverage, and acetabular depth were included in the right side’s follow-up analysis. (Table 4).

**Table 3:** Logistic regression analysis for coxa valga (left side)

Factor	Univariate analysis			Multivariate analysis		
	OR	95%CI	*P* value	OR	95%CI	*P* value
Age (≥40/30–39/< 30)	1.159	0.703, 1.913	0.563			
Gender (male/female)	1.159	0.435, 1.463	0.465			
Weight (kg)	0.992	0.970, 1.015	0.492			
Height (cm)	0.993	0.958, 1.028	0.684			
BMI (> 25/18.5–25/< 18.5)	1.014	0.606, 1.699	0.957			
GMC (+/−)	3.309	1.463, 7.482	0.004	3.253	1.420, 7.449	0.005
LCEA (> 40/25–40/< 25)	0.824	0.446, 1.524	0.538			
Tönnis (> 10/0–10/< 0)	1.539	0.906, 2.614	0.111			
FHCI (< 0.75/ ≥0.75)	0.000	0.000, 0.000	0.999			
acetabular depth (< 0.9/ ≥0.9)	1.210	0.358, 4.091	0.759			
SFP angle (> 80/60–80/< 60)	0.848	0.418, 1.720	0.647			
OF ratio (> 1.4/0.7–1.4/< 0.7)	3.277	1.447, 7.421	0.004	3.218	1.393, 7.433	0.006

*OR* odds ratio, *CI* confidence interval, *NSA* Neck–shaft angle, *LCEA* lateral center edge angle, *FHCI* femoral head coverage index, *SFP angle* Sacro-femoral-pubic angle, *OF ratio* obturator foramen ratio, *BMI* body mass index, *GMC* gluteal muscle contracture

**Table 4:** Logistic regression analysis for coxa valga (right side)

Factor	Univariate analysis			Multivariate analysis		
	OR	95%CI	*P* value	OR	95%CI	*P* value
Age (≥40/30–39/< 30)	0.713	0.426, 1.193	0.198			
Gender (male/female)	0.833	0.453, 1.532	0.556			
Weight (kg)	0.983	0.960, 1.006	0.138			
Height (cm)	0.969	0.934, 1.005	0.090			
BMI (> 25/18.5–25/< 18.5)	0.886	0.527, 1.489	0.647			
GMC (+/−)	35.583	4.800, 263.785	0.000	91.240	7.371, 1129.412	0.000
LCEA (> 40/25–40/< 25)	0.657	0.335, 1.289	0.222			
Tönnis (> 10/0–10/< 0)	1.242	0.737, 2.093	0.416			
FHCI (< 0.75/ ≥0.75)	5.355	1.505, 19.055	0.010	4.240	1.030, 17.456	0.045
acetabular depth (< 0.9/ ≥0.9)	2.302	0.815, 6.503	0.116	16.504	1.893, 143.886	0.011
SFP angle (> 80/60–80/< 60)	0.889	0.419, 1.886	0.760			
OF ratio (> 1.4/0.7–1.4/< 0.7)	0.867	0.414, 1.818	0.706			

*OR* odds ratio, *CI* confidence interval, *NSA* Neck–shaft angle, *LCEA* lateral center edge angle, *FHCI* femoral head coverage index, *SFP angle* Sacro-femoral-pubic angle, *OF ratio* obturator foramen ratio, *BMI* body mass index, *GMC* gluteal muscle contracture

After the following multivariate regression analysis using a stepwise method, we identified GMC and OF ratio as independent risk factors for left hip and GMC, femoral head coverage, and acetabular depth for the right hip. Among them, GMC exists as a common risk factor for the left and right sides.

### Multiple linear regression for SFP angle

**Fig. 2:**
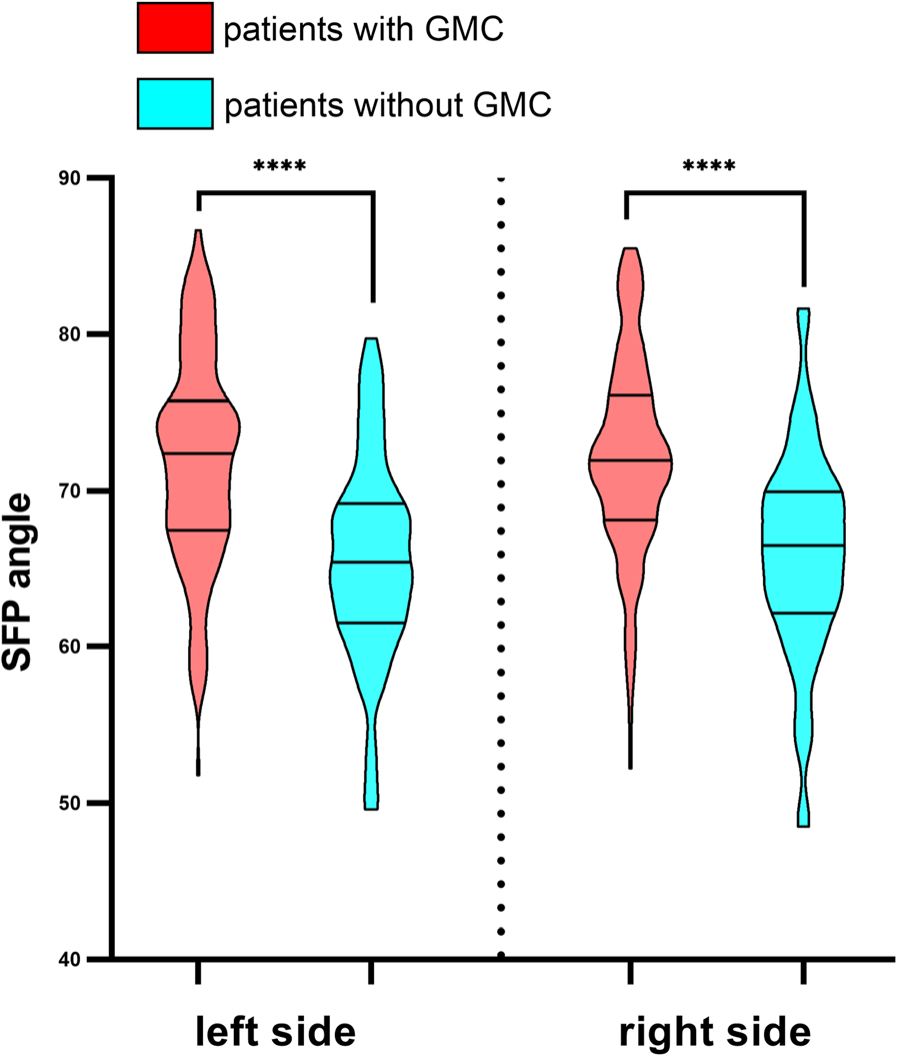
SFP angle of patients with or without GMC. ****, *P* value< 0.0001

The two multiple linear regressions on the left and right revealed that GMC, BMI, LCEA, and acetabular depth are collective influencing factors for both sides’ SFP angle. (Tables 5 and 6) Additionally, a significant difference in SFP angle, as shown in Fig. 2, was found between patients with GMC and without GMC. The mean SFP angle of patients with GMC (71.9 left, 72.2 right) is higher than that of patients without GMC (65.5 left, 65.9 right). These results suggest that there is an association between GMC and higher SFP angle.

**Table 5:** Multiple linear regression analysis predicting SFP angle(left side)

Parameter	β (95% CI)	*P* value
GMC (+/−)	3.987 (2.303, 5.670)	0.000
BMI (kg/m^2^)	−0.424 (−0.633, −.215)	0.000
LCEA	−0.637 (−0.853, −.421)	0.000
FHCI	46.253 (21.844, 70.663)	0.000
Acetabular depth	12.647 (9.661, 15.633)	0.000
OF ratio	−4.735 (−7.611, −1.858)	0.001

*CI* confidence interval, *GMC* gluteal muscle contracture, *BMI* body mass index, *LCEA* lateral center edge angle, *FHCI* femoral head coverage index, *OF ratio* obturator foramen ratio

**Table 6:** Multiple linear regression analysis predicting SFP angle(right side)

Factor	β (95% CI)	*P* value
GMC (+/−)	3.423 (1.842, 5.004)	0.000
Gender (male/female)	− 4.505 (−5.983, −3.026)	0.000
BMI (kg/m^2^)	− 0.284 (− 0.505, − 0.063)	0.012
LCEA	− 0.275 (− 0.405, − 0.146)	0.000
Acetabular depth	13.849 (10.857, 16.841)	0.000

*CI* confidence interval, *GMC* gluteal muscle contracture, *BMI* body mass index, *LCEA* lateral center edge angle

### Characteristics differences between the GMC patients with or without coxa Valga

The chi square test indicated that there was no significant difference(*P* = 0.470) between the incidence of coxa valga in the left and right sides among the GMC patients. In the results of two independent sample t-test, FHCI and OF ratio showed significant differences on both sides, age and acetabular depth showed significant differences only on the right side. (Table 7).

**Table 7:** Characteristics of patients with GMC

	Left side			Right side		
	Without coxa valga (*n* = 50)	With coxa valga (*n* = 102)	*P* value	Without coxa valga (*n* = 56)	With coxa valga (*n* = 96)	*P* value
Age	31.80 [6.68]	30.10 [5.99]	0.116	32.17 [6.49]	29.66 [6.24]	0.02
Gender (male/female)	42/60	22/28	0.740	40/56	24/32	0.89
BMI	21.90 [3.31]	22.04 [2.71]	0.788	21.96 [3.29]	21.93 [2.84]	0.95
LCEA	38.69 [6.82]	36.33 [7.30]	0.052	36.42 [6.40]	34.74 [9.08]	0.23
Tönnis angle	5.56 [5.12]	7.30 [5.82]	0.064	7.58 [4.78]	8.49 [6.46]	0.32
FHCI	0.88 [0.05]	0.85 [0.06]	0.005	0.87 [0.06]	0.84 [0.08]	0.02
Acetabular depth	1.52 [0.28]	1.42 [0.39]	0.102	1.43 [0.27]	1.29 [0.36]	0.01
SFP angle	72.12 [6.63]	71.50 [6.98]	0.594	72.58 [6.35]	71.43 [6.44]	0.29
OF ratio	0.97 [0.24]	1.11 [0.27]	0.003	0.93 [0.24]	1.02 [0.29]	0.03

Data are presented as mean [SD] or number. *SD* standard deviation, *BMI* body mass index, *GMC* gluteal muscle contracture, *LCEA* lateral center edge angle, *FHCI* femoral head coverage index, *SFP angle* Sacro-femoral-pubic angle, *OF ratio* obturator foramen ratio

## Discussion

**Fig. 3:**
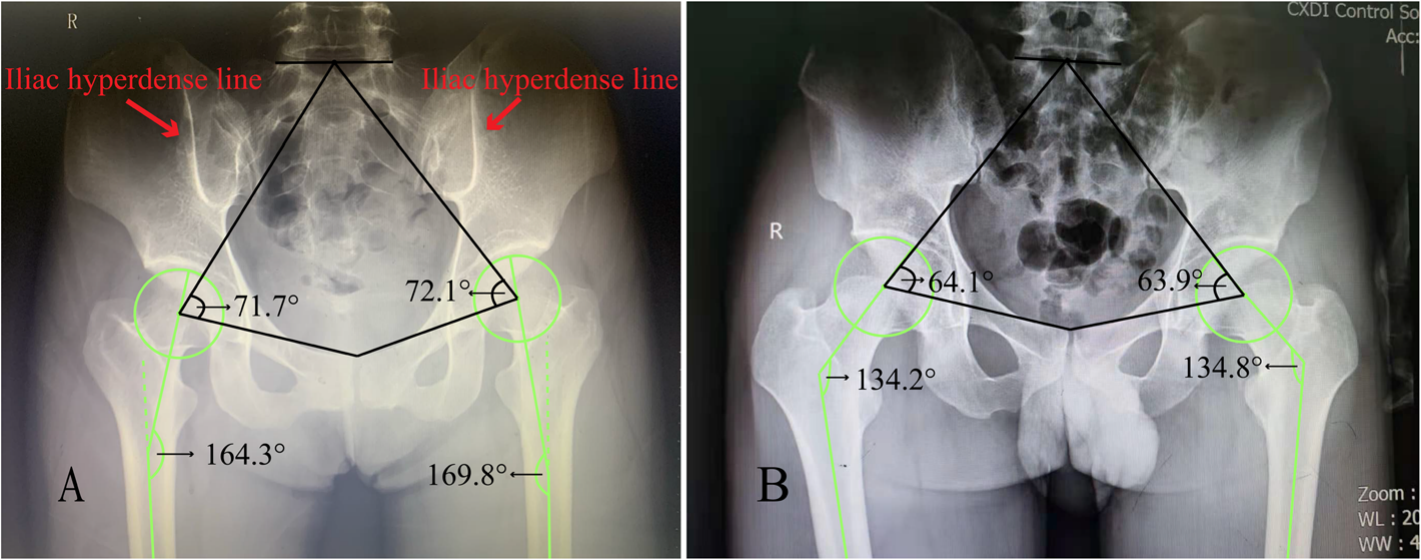
A: AP pelvic radiograph of a patient with GMC; red arrow, iliac hyperdense line. B: AP pelvic radiograph of a patient without GMC

The current study found that GMC is significantly correlated with coxa valga and the increase of SFP angle. A possible explanation for this might be that the onset of GMC in childhood affects hip development. It has been demonstrated that gluteal intramuscular injection with benzyl alcohol, as a solvent for penicillin, in childhood is a significant cause of GMC [7]. As children are in the period of growth and development, the morphology of bone development changes according to physiological needs with the age increase to maintain the balance of normal coordinated development of bones and muscles. The gluteal muscle degeneration and contracture, caused by chemical and physical damage of drug injection, forms a fibrosis band that stretches the pelvis and femoral epiphysis. The skeletal growth in traction direction destroys bones and gluteal muscles’ physiological balance, secondarily affecting the pelvis and femur’s normal morphology. As shown in Fig. 3, AP pelvic radiographs of the representative cases showed marked increases in NSA and SFP angle in the GMC patients compared with the patients without GMC.

The upper outer quadrant of the buttocks, where the gluteus maximus and the anterior fibers of the gluteus medius are located, is a common intramuscular injection site in clinical practice [23]. Therefore, the gluteus maximus and gluteus medius are also the most frequently involved muscles in injection-induced gluteus contracture. A biomechanical study in China [23] showed that although the contractural gluteus muscle still has the characteristics of viscoelastic material, the contracture band’s ultimate strength and elastic modulus are significantly higher than those of the normal gluteus muscle. In contrast, the ultimate strain of the contracture band is significantly lower than that of the normal gluteus muscle. That is to say, the elasticity of the contracture gluteal muscles is greatly reduced on the one hand, and the strength is significantly increased on the other hand.

From the perspective of the local anatomical relationship, the gluteus maximus arises from the posterior gluteal line of the ilium and the rough area of bone, descending laterally and ending at the gluteal tuberosity of the femur and the iliotibial band. Gluteus medius arises between the posterior and anterior gluteal line, ending on the greater trochanter’s lateral surface. Therefore, the contracted gluteal muscles pull outward and downward on the iliac bone, disrupting the pelvis’s force balance, resulting in a forward pelvic tilt (PT). However, limited to the retrospective imaging studies, lateral pelvic radiographs were not obtained to determine the pelvic tilt’s exact degree. Previous studies have proposed SFP angle as devices predicting PT in AP pelvic radiograph [21, 22], and Hu et al. [24] verified the SFP angle’s reliability in estimating PT in Chinese Han nationality adults. In the current study, GMC with higher SFP angle means has lower PT according to the estimation equation (*PT* = 75 − *SFP angle*) [21]. A reduced PT (defined as the angle between the vertical and the line from the center of femoral head to the midpoint of the sacral plate on the lateral pelvic radiograph) means that the pelvis is tilted forward [25]. It can therefore be assumed rationally that the GMC may induce forward pelvic tilt. However, the assumption is based on a forecast, and future research should be undertaken to investigate the relationship between GMC and measured exact pelvic tilt.

On the other hand, since the attachment of the gluteus maximus is located in the gluteal tuberosity of the femur and the iliotibial band, the contracture of the gluteus will produce upward and backward pulling force on the gluteal tuberosity, thus causing the femur abnormal external rotation and extension. The hip joint is relatively stable, and normal individuals do not require gluteus medius muscle contraction to maintain an upright state. However, gluteus medius contracture bands are always under tension due to shortened muscle length and reduced elasticity. Therefore, the anterior fibers of the gluteus medius, positioned almost in the sagittal plane, have persistent traction on the femur in the direction of abduction. Bone development is characterized by reshaping in the direction of traction, as exemplified by the iliac hyperdense line sign, which is a cortical change caused by sustained traction of the gluteus maximus contracture [14].(Fig. 3A) Taken together, the contracture muscles represented by the gluteus maximus and the gluteus medius disrupt the biomechanical balance of the hip joint and generate a sustained outward posterosuperior traction force that induces osseous remodeling. In the long-term, the gluteus muscle contracture eventually leads to abnormal development of the femoral neck and the NSA’s enlargement, thus forming coxa valgus. This study found that there was no correlation between the occurrence of coxa valga and patient age. A possible explanation for this might be that most bones cease growing after reaching adult age.

In the verification of the characteristics differences between the GMC patients with or without coxa valga deformity, this study unexpectedly found that younger patients appeared to be more likely to develop coxa valga (*P* value: 0.116 left and 0.02 right). This result may be explained by the fact that patients with more severe contracture lesions, which are more likely cause coxa valga and earlier symptom, are more likely to seek therapeutic intervention early. In addition, the results of the t-test show that there are significant differences in acetabular depth (*P* value: 0.102 left and 0.01 right), FHCI (*P* value: 0.005 left and 0.02 right) and OF ratio (*P* value: 0.003 left and 0.03 right) between GMC patients with or without coxa valga. A possible explanation for this might be that, as noted previously, patients with more severe contractural gluteal muscles are more likely to have coxa valga while also contributing to deformities of the hip and pelvis. Another possible explanation for this is that patients with coxa valga inherently have genetic or acquired factors for the skeletal deformity. Therefore, future work based on grading the severity of GMC patients is required.

One interesting finding is the difference of risk factors in logistic regression for coxa valga between the left side and right side. There are several possible explanations for this result. Firstly, there is a biomechanical difference between the left hip and the right hip, and the difference in the dominant side can also lead to uneven development. Secondly, there is measurement inaccuracy due to the tilted position of the pelvis. Because the occurrence of GMC is related to the concentration of benzyl alcohol and injection frequency, contracture is more severe on the side with more injection times. Unilateral or bilateral cases with different contracture degrees usually cause uneven force on the pelvis in the coronal plane, resulting in a left or right obliquity of the pelvis while tilting forward. There are similarities between the attitude expressed by Zhang et al. [4], who suggested that GMC can cause leg-length discrepancy and pelvic obliquity. Henebry et al. [26] also demonstrated that pelvic tilt could alter the hip’s radiographic markers in standing AP pelvic radiograph. Thirdly, the changes mentioned above of pelvic tilt and femoral NSA caused by GMC alter the hip joint’s biological stress axis, affecting the acetabulum development and further resulting in the radiographic performance alteration. These alterations including the increase of CE angle and the decrease of acetabular depth and FHCI. The factors existing in GMC change according to the lesion’s degree, and they influence each other, leading to the current results. In future investigations, the severity of GMC should be graded to eliminate confounding factors.

The knee is the most common site of osteoarthritis (OA) in clinical practice. Several risk factors are associated with knee OA, among which skeleton misalignment is one of the inherent risk factors for knee OA [27]. Misalignment of lower extremity changes the normal biomechanical mechanism, thus producing a direct effect on OA progression or indirectly affecting the surrounding tissue [28]. Previous studies have shown that both varus and valgus malalignment of knee joint significantly increases the risk of medial and lateral knee OA, respectively [19, 29–32]. Because the knee joint cannot function independently of the rest of the lower extremity kinematic chain, the hip and ankle’s biomechanical mechanism also significantly affects the knee joint load. Chang et al. [33] showed that, during weight-bearing activity (such as walking), the abnormal gait caused by the limitation of hip abduction could lead to the center of gravity shift and forces acting on the medial compartment cartilage of the stance limb raise, thus increasing the risk of OA. Coskun Benlidayi et al. [34] conducted a study that identified that coxa valga is associated with knee OA severity. The author also set a cut-off value of NSA to predict that people with NSA more than 134.4° have an 8-fold increased risk of severe knee OA [34]. Therefore, coxa valga is a significant risk factor of OA that cannot be ignored.

Additionally, Huang et al. [3] demonstrated that GMC could cause patellofemoral instability (mainly due to iliotibial band contracture, the contracture band often involved in GMC) and knee pain, and arthroscopic release of contracture fibrosis band can significantly alleviate the symptom. It is possible to hypothesize that GMC may result in OA and knee pain by causing coxa valga and patellofemoral instability. Therefore, more attention should be paid to GMC as a significant risk factor of OA and knee pain because it causes coxa valga and induces patellofemoral disease.

A limitation of this study is that patients’ postoperative AP pelvic radiographs were not obtained due to patient wishes and the patients’ own considerations about time and cost. However, Huang et al. [3] have found that surgical release of GMC can significantly reduce the tilt and lateral shift of the patella by comparing preoperative and postoperative knee CT images. Additionally, multiple studies [3, 5, 9, 12, 35, 36], from the functional and symptomatic perspective, have shown that early surgery effectively reduces patient symptoms and improves the quality of life, with high patient follow-up satisfaction. It is reasonable to assume that early diagnosis and surgery are beneficial to patients, while it also needs to be validated by further studies based on comparison of preoperative and postoperative radiographic parameters of AP pelvic radiograph. Another limitation of the current study is that the GMC patients admitted to this department were almost all injection-induced, failing to consider other risk factors, including cerebral palsy. Cerebral palsy as a significant contributor to musculoskeletal disorders requires future work to explore its association with GMC and coxa valga.

## Conclusion

The most prominent finding to emerge from this study is that GMC is a significant risk factor of coxa valga. GMC also increases the SFP angle, so it can be assumed that GMC causes the pelvis to tilt forward, although further studies are needed to verify it. An abnormal hip anatomical relationship can change the biomechanical mechanism and induce OA. Therefore, GMC should be paid attention to and treated early.

## Data Availability

The datasets used or analyzed during the current study are available from the corresponding author on reasonable request.

## Abbreviations

GMC: Gluteal muscle contracture
AP: Anteroposterior
NSA: Neck–shaft angle
LCEA: Lateral center edge angle
FHCI: Femoral head coverage index
SFP: Sacro-femoral-pubic
OF ratio: Obturator foramen ratio
